# Effect of Aerobic and Anaerobic Exercise on the Complement System of Proteins in Healthy Young Males

**DOI:** 10.3390/jcm9082357

**Published:** 2020-07-23

**Authors:** Dorota Kostrzewa-Nowak, Joanna Kubaszewska, Anna Nowakowska, Robert Nowak

**Affiliations:** Centre for Human Structural and Functional Research, University of Szczecin, 70-240 Szczecin, Poland; jo.kubaszewska@gmail.com (J.K.); anna.nowakowska@usz.edu.pl (A.N.); robert.nowak@usz.edu.pl (R.N.)

**Keywords:** healthy men, inflammation, progressive effort, endurance test, speed test

## Abstract

This study was aimed at examining the impact of common types of physical efforts used to determine the aerobic and anaerobic performance of the participants on the complement system in their peripheral blood. Fifty-one physically active young males aged 16 years old (range 15–21 years) were divided into two age groups (younger, 15–17 years old and older, 18–21 years old) and performed two types of intensive efforts: aerobic (endurance; 20-m shuttle run test; Beep) and anaerobic (speed; repeated speed ability test; RSA). Venous blood samples were collected before and after each exercise (5 and 60 min) to profile the complement system components, namely the levels of C2, C3, C3a, iC3b, and C4. The endurance effort caused a decrease in the post-test C3 (*p* < 0.001 for both age groups) and increase in post-test C3a (*p* < 0.001 and *p* < 0.01 for the younger and older group, respectively), recovery iC3b (*p* < 0.001 and *p* < 0.05 for younger and older group, respectively), recovery C2 (*p* < 0.01 for both age groups), and post-test C4 (*p* < 0.05 and *p* < 0.01 for the younger and older group, respectively) levels, while the speed effort caused a decrease only in the post-test C2 (*p* < 0.05 for younger participants) and post-test C4 levels (*p* < 0.001 and *p* < 0.01 for the younger and older group, respectively) and an increase in the recovery C3a level (*p* < 0.05). Our study provides evidence that different types of physical effort promote different immune responses in physically active young men. Aerobic exercise induced the activation of an alternative pathway of the complement system, whilst the anaerobic effort had little influence. A better understanding of the post-exercise immune response provides a framework to prescribe physical activity to achieve different health outcomes.

## 1. Introduction

The complement system is part of the innate immune system, which provides a protective mechanism against pathogens in the absence of specific adaptive immunity [1]. It is a link between the innate and acquired immune systems [2]. Proteins of the complement system are key factors in providing host surveillance and protection through various functions, including targeting inflammatory reactions, phagocyte attraction by chemotaxis, the removal of immune complexes (the scavenging of necrotic and apoptotic debris), activating cells, participating in developmental and regenerative processes, and the modulation of humoral and cell-mediated immune responses [3,4]. The complement system consists of 50 serum and cell surface proteins, which constitute approximately 15% of the globulin fraction, giving more than 3 g/L of protein [2,5].

Each feature of the complement system fulfills a specific role in immunity and is activated by different stimuli. Complement C3 plays a key role in a classic and alternative way of activation. A deficiency of C3 leads to impaired work of the immune system, which leads to increased susceptibility to infection [6]. One of the stages of a well-functioning cascade is the cleavage of the C3 protein into the C3a and C3b components, which is achieved by the C3 convertase known as C4b2a, which also occurs in the lectin activation pathway. In the classical way, the C4 component is cleaved by C1s into two components: C4a and C4b. C4b binds to the cell membrane and connects to C2, which is cleaved into two further subunits, C2a and C2b. Due to fusion of the two C4b and C2a components, a heterodimer called classical-C3 convertase is formed with proteolytic properties, due to the serine protease activity of the C2a element [7,8]. The alternative pathway dominates quantitatively over the classical one [7]. An overactivation of complement activity or incorrect localization can be harmful to the body, leading to serious diseases, including multiple sclerosis, Alzheimer’s disease, asthma, sepsis, or hyperacute organ rejection [7].

One factor influencing the activation of the complement system is physical effort, which is a natural stimulus affecting the defense and immune mechanisms in both specific and cellular and humoral immunity. This interplay is complex, as regular exercise of moderate intensity can have a stimulating effect on the immune system. Conversely, repeated high-intensity exercise (with insufficient recovery), as performed regularly by athletes, can suppress the immune system and increase susceptibility to infections [9,10,11,12]. Intense physical effort can have strong metabolic effects, increasing oxidative stress, the release of heat shock proteins, catecholamines, cortisol, and insulin-like growth factor 1 (IGF-1) [9,13], all of which might contribute to immune stimulation or suppression, depending on other co-factors (e.g., age, fitness level).

The role of the complement system in primary immunodeficiencies, or in defining disease activity in systemic autoimmunity, is well described [4,14,15,16]. However, little research has investigated this system in relation to the acute post-exercise immune response. Knowing that the complement also plays an important role in adaptive immunity involving T and B cells [17,18], but is also involved in tumor growth [19] and human pathological states such as an atypical hemolytic uremic syndrome, age-related macular degeneration [20], and especially in tissue regeneration [7], it seems that this system must be involved in post-effort immune response. In fact, there are works evidencing its involvement in post-effort immunity [21,22,23]. Combining knowledge of T cells in post-effort immune response [24,25,26,27,28,29] and the contribution of the complement system to the activation and differentiation of T cells, as well as maintaining immunological memory, could bridge the gap between immunomodulation and immunodepression following an acute bout of intensive exercise. This itself provides a framework to better explain the “open window theory” [30].

The main aim of this study was to examine the impact of two forms of exercise that differ in physical effort on the complement system and post-exercise immune response in young healthy males. To achieve this, the participants performed two bouts of high-intensity exercise (endurance/aerobic and anaerobic) with blood levels of C2, C3, C3a, iC3b, and C4 compared within and between exercise treatments.

## 2. Materials and Methods

### 2.1. Participants

Fifty-one young physically active males aged 16 years old (range 15–21 years) were recruited and divided according to their age into two groups: younger (15–17 years old) and older (18–21 years old). The participants reported at least 100 min of physical activity per day, with a median training volume equal to 115 min and a median VO_2_max equal to 54.62 mL/kg/min. For study inclusion, the following criteria were employed: no history of any metabolic syndrome or cardiovascular diseases, and no medical history of endocrine or immune disorders. All participants were non-smokers and refrained from taking any medications or supplements (except for protein supplements as reported by some of the participants) before the study commenced. The participants and their parents or guardians, when appropriate, were fully informed of any risks and benefits of the experimental procedures before giving their written consent to participate. This study was approved by the Local Ethics Committee at the Regional Medical Chamber in Szczecin (no. 05/KB/VII/2019) in accordance with the Helsinki Declaration.

### 2.2. Experimental Protocols

Prior to exercise testing, participants’ body mass, body mass index (BMI), basal metabolic rate (BMR), percentage of fat (FAT), fat mass (FAT MASS), and total body water (TBW) were determined using a Body Composition Analyzer (Tanita BC-418MA, Tokyo, Japan). All participants performed two types of physical efforts: endurance and speed-based. A maximal multistage 20-m shuttle run test (Beep test) [31,32] was performed for endurance testing. For speed effort, a repeated speed ability test (RSA test) [33,34] was conducted.

All participants performed both tests (aerobic—Beep test—and anaerobic—RSA test) that started with a standardized warm-up consisting of running at a speed of 5 km/h for 10 min. There was 7 days between each test.

The Beep test (maximal multistage 20-m shuttle run test) was performed indoors (athletics hall) at a temperature of 20–23 °C, two hours after light breakfast. Following standard protocols [31,32], the participants covered 20-m sections in a shuttle format (running back and forth) over several levels of increasing intensity. Each level lasted 60 s in a progressively increasing (by 0.5 km/h) pace, as determined by an audible cue with correspondingly shorter intervals. The test started at a speed of 8.5 km/h. Participants were required to touch their foot (at the 20-m mark) before the signal sounded. It was acceptable to make up any delay in the next 20-m distance. Each participant was asked to stop after two consecutive failed attempts.

Maximum oxygen uptake (VO_2_max) was calculated after the Beep test was calculated according to Flouris et al. formula [35] as follows:

$${\mathrm{VO}}_{2}\max\;\left(\mathrm{mL}/\min/\mathrm{kg}\right)=\left(\max.\;\mathrm{attained}\;\mathrm{speed}\;\left(\mathrm{km}/h\right)\;\times \;6.65-35.8\right)\;\times \;0.95+0.182.$$

The RSA test was conducted in the morning on a 400 m-long athletics track with an ambient temperature of 20–23 °C [33,34], two hours after light breakfast. This test consisted of 10 × 15 m sprints starting every 30 s, with a slow walk (active recovery) between repetitions. Participants were instructed to assume the ready position 5 s before starting the next sprint.

### 2.3. Blood Testing

During each test of physical effort, blood samples were collected at three time points from the cubital vein: before testing (pre-test), no longer than 5 min after exercise (post-test), and about 1 h later, at the end of the lactate recovery period [36,37,38]. At each time point, venous blood samples were collected in a 7.5 mL S-Monovette tube for serum separation (SARSTEDT AG & Co., Nümbrecht, Germany). All analyses were performed immediately following blood collection and serum separation.

The biochemical tests were carried out using an Automatic Clinical Chemistry Analyzer (BM-100, BioMaxima S.A., Lublin, Poland). The blood serum component was tested for the concentration of the following analytes. Albumin, total protein (TP), and C-reactive protein (CRP) concentrations were determined using a colorimetric assay kit (BioMaxima S.A., Lublin, Poland) according to manufacturer’s protocol. Lactic acid (LA) concentration was determined with the use of a colorimetric assay kit (PZ Cormay S.A., Łomianki, Poland) according to the manufacturer’s protocol. C3 and C4 complement components’ concentrations were determined using a colorimetric assay kit (QuimicaClinicaAplicada S.A., Amposta, Spain) according to the manufacturer’s protocol. All the analyses were verified using a multiparametric control serum and a control serum of a normal level (BioNorm) and a high level (BioPath) (BioMaxima S.A., Lublin, Poland).

Enzyme-linked immunosorbent assay (ELISA) kits were used to determine plasma levels of C2 (Cloud-Clone Corp., Katy, TX, USA), and C3a and iC3b (Quidel Corporation, Athens, OH, USA) according to the manufacturers’ protocols. All ELISA tests were performed using a high-throughput microplate reader Synergy H1 (BioTek Instruments, Inc., Winooski, VT, USA).

To compensate for the changes in analyzed blood parameters induced by the exercise test, plasma volume loss (ΔPV) and subsequent correction of those parameters for ΔPV were calculated according to Dill and Costill, and Alis et al.’s equations [39] as follows:

$$∆PV\left(\%\right)=100\times \left(\frac{Hb_{pre}}{Hb_{post}}\times \frac{100-Htc_{post}}{100-Htc_{pre}}-1\right)$$

where Hb_pre_—hemoglobin pre-test (g/dL), Hb_post_—hemoglobin post-test (or in recovery) (g/dL), Htc_pre_—hematocrit pre-test (%), Htc_post_—hematocrit post-test (or in recovery) (%).

The formula for the correction of blood parameters was as follows:

$$\left[Corrected\;parameter\;concentration\right]=\left[Uncorrected\;parameter\;concentration\right]\times \left(1+\frac{∆PV\left(\%\right)}{100}\right).$$

### 2.4. Statistical Analyses

All data are presented as median values (interquartile range), except for age, which is presented as median (minimum–maximum range). The normality of the data was assessed using Shapiro–Wilk test. As a result of non-normal data distribution, non-parametric statistical tests were used. The significance level of differences observed between analyzed time points (pre-exercise versus post-exercise versus recovery) was calculated using Friedman’s analysis of variance for repeated measures followed by post-hoc Dunn’s test with Bonferroni correction. The significance level of differences in analyzed parameters between the Beep and RSA tests or between younger and older groups was calculated using the Mann–Whitney U-test. Each time, *p* < 0.05 was considered as a significant difference. Statistical analysis was performed using Statistica version 13 software (2017; TIBCO Software Inc., Palo Alto, CA, USA).

## 3. Results

Raw data obtained during the study are presented in Supplementary Material Table S1. The participants’ physical characteristics are presented in Table 1.

The results of the tests performed by the participants are presented in Table 2. There were no significant differences between the younger and older group of participants, regardless of their physiological maturation.

The two exercise interventions caused a significant change in the total protein (TP) and albumin levels corrected for plasma volume loss. Both parameters were significantly higher in post-effort time points in comparison to baseline values with the exception of older participants performing the Beep test (Table 3). A significant alteration in CRP level in comparison to the pre-test values was observed only in the younger participants (Table 3). The direction of post-effort and recovery changes in our measured parameters (across both exercise tests) are presented as Δ values, where Δp denotes differences between post-test and pre-test values, and Δr denotes differences between recovery and pre-test values (Table 3).

To confirm the effectiveness of LA recovery, LA level was monitored, and appropriate Δ values were calculated (Table 3).

After the Beep test, all complement components differed significantly from baseline values regardless of the age of participants, while after the RSA test, the changes in complement components were significantly different from the pre-test data only in the younger group of participants (Table 4). After the Beep test, the post-test decrease in C3 level is in line with C3a and the functional form of iC3b proteins. Only the iC3b level is significantly lower than the pre-test values during LA recovery, after the Beep test, while the level of C3 at this time point was significantly lower only in the older group of participants (Table 4). Only a decrease in C4 level emerged with the RSA test among older participants (Table 4). The parameter changes were also described as Δp and Δr changes, thereby providing information about the direction and magnitude of change (“+” or “–”) (Table 4).

To help the reader compare our results with data provided by other groups, the biochemical variables and complement variables of studied participants’ blood samples are presented as mean ± SD in Supplementary Material Tables S2 and S3, respectively.

**Figure 1:**
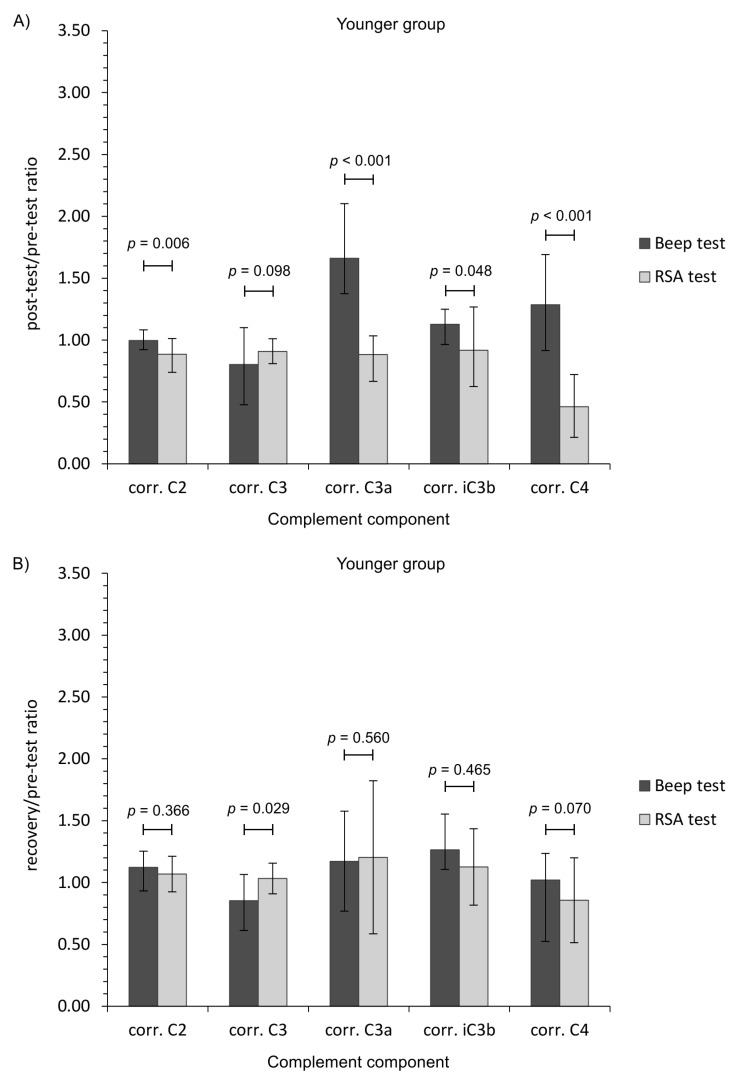

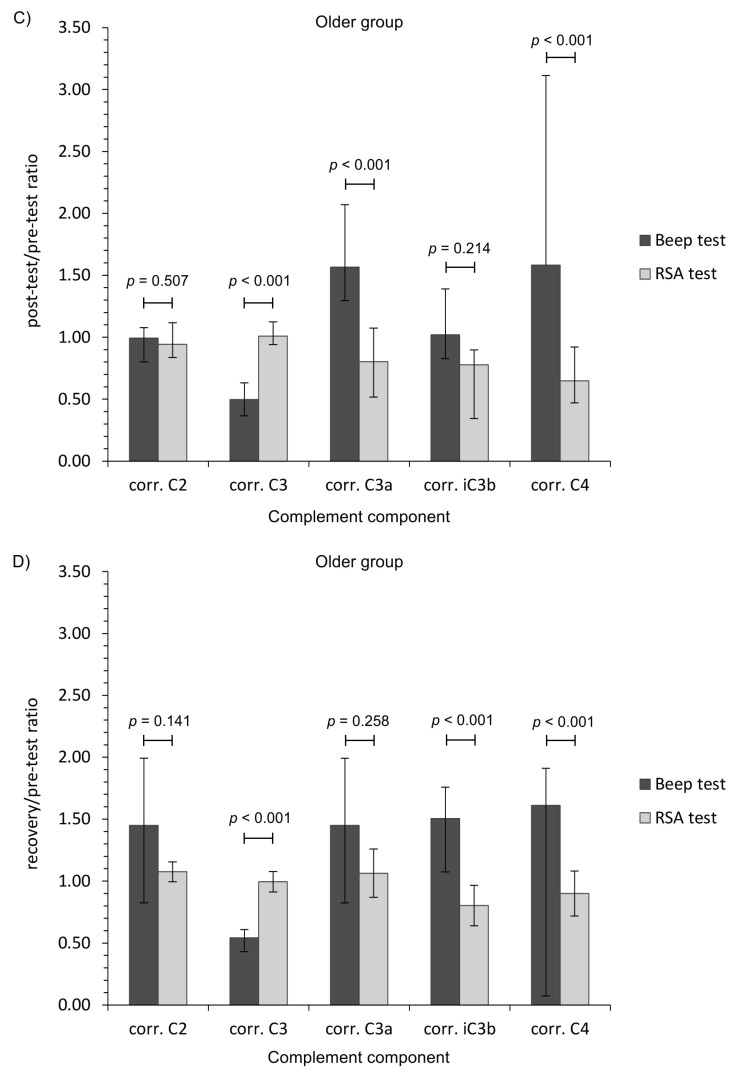
Ratio measures of studied complement component levels corrected for plasma volume loss after the Beep test and RSA test among studied participants. (**A**,**C**) Post-test/pre-test values; (**B**,**D**) recovery/pre-test values. The data are presented as median and Q1–Q3 range. Beep—maximal multistage 20-m shuttle run test, corr.—appropriate values corrected for plasma volume loss, RSA—reaped speed ability test.

To add clinical value, we also calculated ratio measures of complement component levels (post-test/pre-test, and recovery/pre-test values). This metric revealed additional, and significant, patterns of change between the two types of physical effort (Figure 1).

## 4. Discussion

The role of physical effort as a factor leading to broadly understood changes in the immune system is widely discussed in the literature [9,10,12,40,41,42]. Its influence on the immune system gives heterogeneous biological effects, underlying many different dependencies. On one hand, it is assumed that high-intensity physical effort weakens the organism’s immunity [9,41,42] and their extremely prolonged effect can lead to immunosuppression [9,10]. On the other hand, regular, moderate-intensity physical activity stimulates the formation and increase in immunity [9,41]. One of the symptoms of short-term physical exercise is leukocytosis, resulting from the redistribution of tissue cell components into the blood [43,44]. Disturbances in the functioning of cellular components, such as T lymphocytes or natural killer (NK) cells, are associated with the frequency of high-intensity short-term exercise. This leads to the shift of balance toward an initiation of inflammation, including the secretion of pro-inflammatory and regulatory (anti-inflammatory and multifunctional) signaling factors, causing a violent and aggressive inflammatory response that resembles the immune response to primary antigens [43,45]. Regular, moderate-intensity exercise is one of the most important factors delaying the senescence of the immune system [43,46]. Prolonged exercise can have a pleiotropic effect on the immune system. The intensity of effort depends to a large extent on how it will affect the immune system. Intensive physical effort is associated with the activation of cellular components and their rapid redistribution [10,42,47,48]. It can be assumed that short-term physical exertion with an intensity <60% VO_2_max does not lead to the mobilization of the immune system, but rather, it has modulating effect [9,49] as opposed to high (>70% VO_2_max) and very high (>90% VO_2_max) intensity that may contribute to lowering the athletes’ immunity [9,43,44].

The general finding was that aerobic (Beep test) and anaerobic (RSA test) efforts had a differential impact of the complement system in young physically active men. Aerobic exercise induced the activation of an alternative pathway of the complement system, whilst the anaerobic effort had little influence, although there were differences regarding the age of participants. The calculated ratio measures provide further evidence that each form of exercise presents a markedly different stress stimulus (Figure 1). Moreover, only the RSA test caused CRP corrected for plasma volume loss level to increase, while TP and albumin changes were related with post-effort dehydration before a return to baseline values during recovery (Table 3) [50,51]. On the other hand, the lack of a significant decrease in C2 and C4 protein level following the Beep test lends support to an alternative pathway of complement activation [8]. It is unclear why the C2 protein level continued to rise at each time point after the Beep test, but these observations are in line with the biological response (to a similar physical effort) in the volleyball players [52].

The significant decrease in C4 and C3 proteins is a common observation in both athletes and non-athletes [21,22,52,53]. Interestingly, a significant decrease in C4 level after the RSA test was not in line with C3 activation. No significant changes in C3 level, and its activated forms C3a and iC3b, were demonstrated with RSA testing among older participants at any time after exercise. This may suggest that the signal form C4 protein was not strong enough to activate the complement system. Karacabey et al. [21,23] found that both aerobic exercise on a treadmill for 30 min and a Wingate (anaerobic) test for 30 s caused a significant decrease in C4 and C3 proteins. Interestingly, only the C3 level in Karacabey et al.’s was comparable with the data for the older group performing aerobic exercise (e.g., 178±6 and 104 ± 3 mg/dL for pre-exercise and post-exercise, respectively in Karacabey et al. versus 198±17 and 99 ± 32 mg/dL in our study, when providing mean ± SD) [23]. However, it must be emphasized that our data are provided as corrected for plasma volume loss that was not provided by Karacabey et al. [23]. The exercises, although being aerobic and anaerobic, were also different between their study and ours. Mashiko et al. [22] reported a similar post-game observation in rugby players. In contrast to our findings for the Beep test, the levels of C3 and C4 after short-term maximal exercise rapidly returned to baseline values [40,54], but they stayed slightly lowered after an ultramarathon [55]. When analyzing the runners, Smith et al. reported that short-term aerobic exercise triggers the activation of C3 and C4 complement components and subsequent increase in C3a and C4a [56]. They suggest that regularly engaged aerobic exercise may cause activation of the classical pathway of complement activation as well as a selective downregulation of C3 synthesis [56]. It is clear that C3 and C4 are proteins that depend on the time of effort application. Berk et al. [57] showed that the C3 basal values are lowered more by long-lasting physical effort than an intermittent one. They also indicate that C4 values are higher in intermittent exercises than in running [57]. However, they did not examine the activated forms of those proteins (e.g., C3a, C4a). Navarro Sanz et al. observed a significant increase in C3 and C4 levels after intermittent bouts of an 800 m run at a maximal speed with 30 s of recovery in between [58]. Semple et al. analyzed complement components in the cyclists taking part in Vuelta a España [59]. However, their studies were conducted across wide time ranges, since they determined these proteins in two points after an accumulated distance of about 1200 km. They observed no changes in C3 and an increase in C4 but only on the 11th day and not on the 21st day of the race [59]. On the other hand, an 8 mile-long (12.8 km) training run at 70–75% VO_2_max did not influence the C3 and C4 level both 10 min and 24 h after the exercise [60]. Nieman et al. [40] studied marathon runners and their sedentary counterparts performing graded exercise on a mechanical treadmill. They observed a post-effort increase in C3 and C4 complement components in both studied groups [40]. A similar trend was observed in the case of C4 in older participants performing aerobic exercise in our study. On the other hand, they speculate that these changes were caused by plasma volume reduction [40]. It is known that graded exercise on mechanical treadmill performed by Nieman et al.’s participants does not reflect the physiological demands of a maximal multistage 20-m shuttle run test (Beep test). However, both tests are examples of aerobic exercise. A significant increase in post-exercise C4 level was observed by Cordova et al. after an incremental maximal cycling test using a mechanically braked cycle ergometer [61]. It is in line with our observation in regard to older participants performing aerobic exercise (Beep test).

A significant increase in iC3b component concentration, being liberated during the conversion of opsonin C3b [4,62], during lactate recovery (versus pre-test values) regarding the age of participants suggests that aerobic exercise using the 20-m shuttle (Beep) test activates C3 convertases. On the other hand, the iC3b fragment, although bound to the cell surface yet unable to form convertase, plays an important role in signal transduction. It binds to complement receptors on immune cells; therefore, it is an important component of the defense system and homeostasis [4]. From this viewpoint, it seems that the endurance effort (Beep test) exerts an immunomodulatory effect among young physically active men. This observation is in line with our previous study describing the impact of endurance-type exercise on T cells in the peripheral blood of young soccer players [24,25,27].

The most probable explanation of complement system activity, in the post-effort immune response, is the restoration of homeostasis after high physical effort, which signals cell death pathways in the peripheral cells, as seen in elite athletes [26,63] and firefighters [63]. In turn, this enables the damaged cells to be opsonized before being phagocytized by leukocytes [7,8,64]. Artero et al. [65] examined the correlation between muscular fitness and inflammatory parameters, including C3 and C4 complement components, among adolescents. One of the parameters defining health-related fitness was a 20-m shuttle run test (Beep test). They concluded that C3 and C4 levels significantly inversely correlate with the Beep test results [65].

Knowledge of the complement system, in terms of its response trajectory to acute high-intensity exercise, can provide insight regarding mechanisms of exercise immunology and potentially provides a stronger molecular basis for the prevention of cardiovascular diseases (CVD). According to Blankenberg et al. [66], the recruitment of inflammatory cells takes place in CVD impairment and thus, they are candidate particles of higher importance in predicting future CVD events than current risk factors including tobacco smoking, physical inactivity, unhealthy diet, and alcohol abuse. It is known that all-cause and especially CVD mortality negatively correlate with both cardiorespiratory and muscular fitness levels in adults [67,68]. The research on CRP as the marker of inflammatory status regarding physical activity confirms the anti-inflammatory effects of this protein [50,69]. In another work [69], Phillips et al. found that C3 concentrations were positively associated with increasing sedentary behavior and negatively associated with increasing moderate to vigorous physical activity. The level of inflammation markers, calculated as a C3/C4 level ratio [53] in our study, indicated that after the Beep test, there was a decreasing C3/C4 ratio (pre-test value 11.8; post-test: 8.4 and recovery: 9.2 in the younger group and 57.9, 11.6, and 11.8 in the older group, respectively), while after the RSA test, the C3/C4 results differed at corresponding time points (16.0, 25.6, and 21.0 in the case of younger participants and 8.9, 13.9, and 9.0 in the older group, respectively). These results are congruent with the CRP fluctuations observed herein. Delgado-Alfonso et al. [53] also found significant differences in C3 and other inflammatory biomarkers between adolescents who have different physical fitness levels.

## 5. Conclusions

Literature data describing the impact of physical effort on complement system activation are not numerous, and the results presented in them are not consistent. Our study attempted to examine the impact of two types of high-intensity physical exercise, generally described as being aerobic and anaerobic in nature on participants’ complement systems. We present evidence that each type of effort caused different immune responses in physically active young men regarding the complement system. Knowing that the complement system takes part in the activation and differentiation of T cells, as well as maintaining immunological memory and that different T cell subsets are altered in post-effort immune response depending of the type of exercise [24,25,26,27], it could lead to the speculation that aerobic and anaerobic exercise may have different types of impact on the post-effort susceptibility to upper respiratory illness, which is described as “open window theory”. It may be explained by different molecular mechanism with the participation of the complement system.

Regarding the limitations of the study, it was performed on a limited number of participants and only males. One of the reasons was to avoid entering another variable, namely the possible influence of hormone changes during women’s menstrual cycles. However, adding a group of women would significantly enrich future studies. The study group consisted of well-trained participants to ensure group homogeneity regarding participants’ fitness level, especially VO_2_max values. However, including less trained or even sedentary participants would give a broader perspective of the influence of aerobic and anaerobic effort on the complement system. Moreover, the analysis of the anti-inflammatory system and cortisol level would give some more perspective on the crosstalk between complement components, as well as inflammatory proteins and the anti-inflammatory response of athletes to a given exercise bout.

Another limitation is the lack of standardized diet for the participants. It was intended so as to not provide any additional stress related to changing the diet, and the participants were asked to keep their daily routine regarding the diet. However, providing a dietician-consulted diet before and during more extensive research would enrich the study.

## Figures and Tables

**Table 1:** Participants’ body composition variables and VO_2_max values.

Variable	Younger Group (*n* = 39)	Older Group (*n* = 12)	p_MW_^1^
Age (years)	16 (15–17)	19 (18–21)	<0.001
Height (cm)	180 (177–185)	182 (180–184)	0.189
Weight (kg)	70.2 (64.0–75.3)	77.3 (72.9–79.2)	0.010
BMI (kg/m2)	21.7 (20.0–22.7)	22.6 (21.9–23.9)	0.008
BMR (kJ)	8004 (7636–8933)	8771 (8219–9016)	0.229
Fat (%)	12.5 (9.5–14.6)	8.6 (6.5–12.7)	0.060
Fat mass (kg)	8.6 (6.2–9.9)	6.5 (4.6–9.4)	0.296
FFM (kg)	59.9 (55.6–66.4)	67.4 (64.4–69.8)	0.010
TBW (kg)	43.9 (40.7–48.6)	49.3 (47.1–51.1)	0.011
VO_2_max (mL/kg/min)	54.62 (51.46–57.78)	57.78 (53.04–57.78)	0.256

The table presents median (interquartile range), except for age, which is presented as median (minimum–maximum range) values characterizing the participants. BMI—body mass index, BMR—basal metabolic rate, FFM—fat-free mass, TBW—total body water, VO_2_max—maximal oxygen uptake, *n*—number of participants. ^1^ Differences observed between analyzed groups (younger group vs. older group) were assessed using the Mann–Whitney U-test.

**Table 2:** The results of the tests performed by the participants.

Test Result	Younger Group (*n* = 39)	Older Group (*n* = 12)	p_MW_^1^
Beep decimal score	12.7 (10.1–15.3)	13.7 (10.7–15.0)	0.257
RSA mean score (s)	3.28 (3.12–3.55)	3.22 (3.10–3.37)	0.271

The table presents median (minimum-maximum range) values characterizing the participants’ tests performance. ^1^ Differences observed between analyzed groups (younger group vs. older group) were assessed using the Mann–Whitney U test. RSA—repeated speed ability test.

**Table 3:** Selected biochemical variables of studied participants’ blood samples.

Variable		Younger Group (*n* = 39)		Older Group (*n* = 12)	
		Beep Test	RSA Test	Beep Test	RSA Test
Corrected TP (g/L)	pF ^1^	0.009	<0.001	0.338	0.002
	pre-test	66.3 (64.2–68.6)	69.7 ^aa^ (67.7–72.1)	66.6 (64.2–68.2)	70.8 ^aa^ (68.9–72.8)
	post-test	64.4 ^bb^ (61.3–70.0)	67.1 ^bbb^ (64.3–69.2)	66.7 (61.5–68.2)	65.7 ^b^ (63.0–67.6)
	recovery	67.5 (64.7–73.2)	70.9 (68.1–77.0)	68.2 (63.3–71.0)	68.7 (66.1–72.8)
Δ corrected TP	Δp	3.4 (1.9–5.0)	–2.2 (−4.7–−0.3)	−1.6 (−4.3–1.6)	−4.0 (−6.9–−2.2)
	Δr	1.5 (−1.5–4.9)	0.2 (−2.3–8.3)	2.1 (−2.8–4.6)	−1.1 (−5.6–3.6)
Corrected albumin (g/L)	pF	<0.001	<0.001	0.338	0.009
	pre-test	47.3 (46.2–49.3)	52.2 ^aa^ (49.6–54.4)	47.5 (46.0–48.1)	52.9 ^aa^ (51.8–55.1)
	post-test	46.0 ^bbb^ (42.3–48.4)	49.7 ^bbb^ (47.7–50.7)	46.1 (44.2–47.2)	48.2 (46.8–49.0)
	recovery	50.0 ^c^ (46.5–52.1)	52.7 (49.6–55.5)	48.5 (44.7–50.2)	49.5 (47.7–52.9)
Δ corrected albumin	Δp	−2.0 (−4.5–0.9)	−2.3 (−4.9–0.4)	−2.0 (−3.0–0.7)	−6.0 (−7.4–−2.8)
	Δr	1.6 (−1.1–5.3)	−0.3 (−4.0–7.0)	1.5 (−1.6–2.3)	−4.8 (−6.1–0.3)
Corrected CRP (mg/L)	pF	<0.001	0.003	0.039	0.779
	pre-test	1.50 ^aaa^ (1.00–2.00)	3.91 ^aa^ (3.25–4.50)	1.15 (0.60–2.00)	4.89 (4.26–6.16)
	post-test	0.32 (0.00–0.83)	4.65 (4.08–6.85)	1.18 ^b^ (0.84–1.71)	5.72 (3.97–6.13)
	recovery	0.20 ^cc^ (0.00–1.22)	4.39 ^c^ (2.69–7.37)	1.94 (1.44–2.30)	5.00 (4.53–6.25)
Δ corrected CRP	Δp	−1.10 (−1.80–−0.15)	0.85 (0.04–2.56)	−0.13 (−0.50–0.43)	0.29 (−048–0.96)
	Δr	−1.30 (−1.80–0.17)	0.30 (0.06–2.26)	0.72 (−0.15–1.26)	0.09 (−1.34–1.10)
Corrected LA (mmol/L)	pF	<0.001	<0.001	<0.001	<0.001
	pre-test	3.1 ^aaa^ (2.8–3.5)	5.3 ^aaa^ (3.0–6.2)	3.2 ^aa^ (2.9–3.5)	6.1 ^aaa^ (5.4–7.3)
	post-test	9.2 ^bbb^ (7.5–10.7)	16.4 ^bbb^ (9.8–20.5)	10.1 ^bbb^ (8.9–11.5)	20.5 ^bbb^ (18.3–21.0)
	recovery	3.1 (2.6–3.6)	4.2 (2.5–5.7)	2.7 (2.5–3.4)	5.3 (4.5–6.2)
Δ corrected LA	Δp	6.1 (4.4–7.6)	10.4 (6.9–15.7)	7.2 (5.5–8.0)	13.8 (11.7–15.3)
	Δr	−0.1 (−0.6–0.2)	−0.5 (−1.0–0.2)	−0.3 (−0.7–−0.1)	−0.6 (−1.3–−0.2)

^1^ Significance levels of differences observed between analyzed time points (pre-test vs. post-test vs. recovery) were assessed using Friedman’s analysis of variance for repeated measures (pF— Friedman’s ANOVA p values) followed by post-hoc Dunn’s test with Bonferroni correction. The table presents median (Q1–Q3) of values corrected for plasma volume loss. Beep—maximal multistage 20-m shuttle run test, CRP—C-reactive protein, LA—lactic acid, RSA—reaped speed ability test, TP—total protein. Δ—the difference between results: Δp = post-test–pre-test, Δr = recovery–pre-test. The analyses were performed before (baseline, pre-test) and after the effort (5 min post-effort and during lactate recovery time about 1 h after the test). Post-hoc p values: ^aa^ *p* < 0.01, ^aaa^ *p* < 0.001 for pre-test vs. post-test, ^b^ *p* < 0.05, ^bb^ *p* < 0.001, ^bbb^ *p* < 0.001, for post-test vs. recovery, ^c^ *p* < 0.05, ^cc^ *p* < 0.01 for pre-test vs. recovery.

**Table 4:** Complement variables of studied participants’ blood samples.

Variable		Younger Group (*n* = 39)		Older Group (*n* = 12)	
		Beep Test	RSA Test	Beep Test	RSA Test
Corrected C2 (ng/mL)	pF ^1^	<0.001	<0.001	0.002	0.097
	pre-test	8.86 (7.69–9.87)	7.25 ^a^ (6.06–8.36)	9.57 (7.92–10.31)	7.57 (6.57–8.12)
	post-test	8.80 ^bbb^ (8.08–9.62)	6.05 ^bbb^ (5.22–8.12)	8.51 ^bb^ (7.82–9.52)	7.00 (6.09–8.08)
	recovery	9.88 ^cc^ (8.92–11.21)	7.95 (6.27–10.0)	11.33 ^cc^ (9.53–13.07)	8.60 (6.92–9.16)
Δ corrected C2	Δp	−0.04 (−0.70–0.68)	−0.81 (−1.67–0.16)	−0.23 (−2.01–0.64)	−0.44 (−2.05–0.31)
	Δr	1.20 (−0.09–2.39)	0.40 (−0.53–1.73)	1.64 (0.43–2.67)	0.45 (−0.38–1.23)
Corrected C3 (mg/dL)	pF	<0.001	0.005	<0.001	0.920
	pre-test	114.2 ^aaa^ (80.8–156.3)	85.0 (74.6–98.6)	192.7 ^aaa^ (189.0–212.9)	79.6 (66.0–99.4)
	post-test	86.0 ^b^ (62.3–111.3)	74.0 ^bb^ (61.6–93.5)	100.0 (68.7–126.2)	85.0 (68.4–88.1)
	recovery	92.9 (74.8–118.6)	91.4 (73.0–103.0)	110.8 ^cc^ (98.2–123.5)	81.2 (67.9–88.3)
Δ corrected C3	Δp	−22.8 (−66.5–7.7)	−8.8 (−16.3–0.6)	−102.2 (−118.5–−76.7)	0.7 (−9.4–6.1)
	Δr	−15.4 (−46.2–9.1)	3.2 (−6.6–14.6)	–89.3 (−110.1–−66.1)	−0.3 (−9.1–4.9)
Corrected C3a (ng/mL)	pF	<0.001	<0.001	0.002	0.174
	pre-test	0.26 ^aaa^ (0.20–0.35)	0.27 (0.19–0.36)	0.22 ^aa^ (0.19–0.27)	0.27 (0.22–0.35)
	post-test	0.43 ^bbb^ (0.36–0.56)	0.27 ^bbb^ (0.16–0.35)	0.39 (0.32–0.46)	0.20 (0.14–0.31)
	recovery	0.31 (0.22–0.40)	0.35 ^c^ (0.27–0.43)	0.37 (0.25–0.42)	0.32 (0.26–0.36)
Δ corrected C3a	Δp	0.19 (0.10–0.27)	−0.04 (−0.09–0.03)	0.15 (0.08–0.21)	−0.04 (−0.10–0.02)
	Δr	0.03 (−0.06–0.13)	0.05 (0.02–0.18)	0.10 (−0.03–0.21)	0.02 (−0.02–0.06)
Corrected iC3b (mg/mL)	pF	0.001	0.855	0.006	0.174
	pre-test	0.70 (0.66–0.87)	0.37 (0.30–0.60)	0.63 (0.54–0.76)	0.46 (0.28–0.58)
	post-test	0.80 (0.71–0.93)	0.35 (0.24–0.45)	0.77 ^b^ (0.44–0.90)	0.36 (0.29–0.47)
	recovery	0.95 ^ccc^ (0.76–1.07)	0.37 (0.30–0.54)	0.94 ^c^ (0.92–1.04)	0.39 (0.28–0.44)
Δ corrected iC3b	Δp	0.10 (−0.03–0.18)	−0.05 (−0.23–0.08)	0.02 (−0.09–0.22)	−0.07 (−0.15–0.06)
	Δr	0.21 (−0.02–0.31)	0.05 (−0.07–0.15)	0.34 (0.19–0.43)	−0.08 (−0.16–−0.01)
Corrected C4 (mg/dL)	pF	0.022	<0.001	<0.001	0.004
	pre-test	9.64 ^a^ (5.22–13.80)	5.31 ^aaa^ (2.13–7.84)	3.33 ^aa^ (2.80–8.46)	8.91 ^aa^ (4.75–11.93)
	post-test	10.21 (7.33–15.72)	2.89 ^bb^ (0.45–6.07)	8.62 (6.20–10.17)	6.13 (2.45–9.30)
	recovery	10.10 (7.24–15.30)	4.35 (1.51–6.93)	9.40 ^ccc^ (5.50–13.55)	9.03 (4.04–12.08)
Δ corrected C4	Δp	1.73 (−1.20–4.77)	−1.87 (−2.99–−1.22)	2.87 (1.81–5.88)	−2.11 (−3.10–−1.56)
	Δr	0.14 (−2.79–4.26)	–0.65 (−2.71–0.74)	4.70 (1.62–6.91)	−0.80 (−1.88–0.77)

^1^ Significance levels of differences observed between analyzed time points (pre-test vs. post-test vs. recovery) were assessed using Friedman’s analysis of variance for repeated measures (pF— Friedman’s ANOVA p values) followed by post-hoc Dunn’s test with Bonferroni correction. The table presents median (Q1–Q3) of values corrected for plasma volume loss. Beep—maximal multistage 20-m shuttle run test, RSA—reaped speed ability test, TP—total protein. Δ—the difference between results: Δp = post-test–pre-test, Δr = recovery–pre-test. The analyses were performed before (baseline, pre-test) and after the effort (5 min post-effort and during lactate recovery time about 1 h after the test). Post-hoc p values: ^a^ *p* < 0.05, ^aa^ *p* < 0.01, ^aaa^ *p* < 0.001 for pre-test vs. post-test, ^b^ *p* < 0.05, ^bb^ *p* < 0.001, ^bbb^ *p* < 0.001, for post-test vs. recovery, ^c^ *p* < 0.05, ^cc^ *p* < 0.01, ^ccc^ *p* < 0.001 for pre-test vs. recovery.

## Supplementary Materials

The following are available online at https://www.mdpi.com/2077-0383/9/8/2357/s1, Table S1: Raw data obtained during the study; Table S2: Selected biochemical variables of studied participants’ blood samples; Table S3: Complement variables of studied participants’ blood samples.
