# Supplementary material for: Effect of Aerobic and Anaerobic Exercise on the Complement System of Proteins in Healthy Young Males
Source: J Clin Med. 2020 Jul 23;9(8):2357. doi: 10.3390/jcm9082357 (PMC7464301; doi:10.3390/jcm9082357)
Supplement: Supplementary file 1 [file jcm-09-02357-s001.zip › jcm-856170_TableS2.pdf]

Table S2. Selected biochemical variables of studied participants' blood samples.

Variable		Younger group (N = 39)		Older group (N = 12)	
		**Beep test**	**RSA test**	**Beep test**	**RSA test**
Corrected TP (g/L)	pre-test	66.6±3.2	69.9±3.2	66.7±3.4	70.0±4.9
	post-test	64.7±6.1	67.2±4.9	65.2±6.0	65.9±3.4
	recovery	68.8±6.7	73.2±10.3	67.5±4.9	69.6±5.0
Δ corrected TP	Δp	-2.0±5.4	-2.7±3.6	-1.5±4.5	-4.1±4.6
	Δr	2.2±6.1	3.3±10.0	0.8±4.4	-0.4±6.6
Corrected albumin (g/L)	pre-test	47.7±2.4	52.0±3.3	47.2±1.5	53.2±2.5
	post-test	45.6±4.2	49.3±3.3	45.7±3.3	48.3±2.5
	recovery	49.9±4.7	53.5±6.9	47.7±3.3	50.6±4.3
Δ corrected albumin	Δp	-2.1±3.8	-2.7±3.7	-1.5±2.9	-4.9±2.9
	Δr	2.2±4.4	1.5±7.6	0.5±2.7	-2.6±5.2
Corrected CRP (mg/L)	pre-test	1.54±0.84	3.98±1.35	1.39±1.09	5.04±1.86
	post-test	0.54±0.66	5.34±2.12	1.31±0.70	5.19±1.33
	recovery	0.65±0.87	5.30±3.05	1.95±0.56	5.26±2.26
Δ corrected CRP	Δp	-0.99±0.98	1.36±1.83	-0.09±0.57	0.16±0.95
	Δr	-0.89±1.15	1.32±2.67	0.56±0.78	0.22±1.92
Corrected LA (mmol/L)	pre-test	3.2±0.7	4.9±1.6	3.2±0.44	6.3±1.2
	post-test	9.2±2.4	15.7±5.9	10.0±2.0	19.9±3.4
	recovery	3.1±0.7	5.3±5.4	2.9±0.6	5.5±1.3
Δ corrected LA	Δp	5.9±2.3	10.8±4.9	6.8±2.1	13.8±3.0
	Δr	-0.1±0.8	0.4±4.9	-0.3±0.8	-0.9±1.0

The table presents mean±SD of values corrected for plasma volume loss. Beep - maximal multistage 20m shuttle run test, CRP – reactive protein C, LA – lactic acid, RSA – reaped speed ability test, TP – total protein. Δ – the difference between results: Δp = post-test – pre-test, Δr = recovery – pre-test. The analyses were performed before (baseline, pre-test) and after the effort (5 minutes post-effort and during lactate recovery time about 1 hour after the test).

It must be pointed out that this form of non-normally distributed data presentation is not suggested as it may be misleading.
