# Supplementary material for: Effect of Aerobic and Anaerobic Exercise on the Complement System of Proteins in Healthy Young Males
Source: J Clin Med. 2020 Jul 23;9(8):2357. doi: 10.3390/jcm9082357 (PMC7464301; doi:10.3390/jcm9082357)
Supplement: Supplementary file 1 [file jcm-09-02357-s001.zip › jcm-856170_TableS3.pdf]

Table S3. Complement variables of studied participants' blood samples.

Variable		Younger group (*N* = 39)		Older group (*N* = 12)	
		**Beep test**	**RSA test**	**Beep test**	**RSA test**
Corrected C2	pre-test	8.86±1.38	7.44±1.87	9.65±2.12	7.78±1.78
(ng/mL)	post-test	8.76±1.36	6.68±2.15	9.54±2.92	7.09±1.05
	recovery	10.10±1.97	8.10±2.05	11.34±1.90	8.06±1.26
Δ corrected	Δp	-0.11±1.28	-0.76±1.13	-0.11±2.74	-0.69±1.62
C2	Δr	1.24±2.20	0.66±1.60	1.69±1.44	0.27±1.65
Corrected C3	pre-test	119.3±50.8	86.9±19.7	197.6±16.7	80.6±17.1
(mg/dL)	post-test	88.0±27.9	79.5±21.6	98.6±32.5	79.7±11.9
	recovery	98.4±38.7	89.5±18.2	108.5±18.3	79.0±12.4
Δ corrected	Δp	-31.3±52.9	-7.4±13.6	-99.0±34.4	-0.9±9.8
C3	Δr	-20.9±50.9	2.6±20.0	-89.1±26.5	-1.6±7.2
Corrected C3a	pre-test	0.47±0.20	0.30±0.17	0.26±0.15	0.28±0.09
(ng/mL)	post-test	0.51±0.18	0.26±0.14	0.40±0.13	0.24±0.14
	recovery	0.33±0.17	0.38±0.16	0.35±0.11	0.31±0.07
Δ corrected	Δp	0.20±0.15	-0.04±0.10	0.14±0.08	-0.04±0.09
C3a	Δr	0.06±0.19	0.07±0.20	0.09±0.17	0.03±0.10
Corrected	pre-test	0.73±0.15	0.43±0.19	0.64±0.17	0.48±0.25
iC3b (mg/mL)	post-test	0.80±0.19	0.36±0.19	0.69±0.25	0.42±0.23
	recovery	0.90±0.26	0.45±0.20	0.94±0.20	0.39±0.16
Δ corrected	Δp	0.07±0.15	-0.07±0.22	0.05±0.19	-0.07±0.20
iC3b	Δr	0.17±0.20	0.02±0.20	0.30±0.20	-0.09±0.14
Corrected C4	pre-test	9.72±5.76	5.36±3.68	5.26±3.54	8.53±5.05
(mg/dL)	post-test	11.28±4.98	3.34±3.20	9.01±3.46	5.82±4.26
	recovery	10.57±5.14	4.53±3.57	9.60±4.76	8.34±4.97
Δ corrected	Δp	1.56±6.09	-2.03±1.55	3.75±2.59	-2.71±2.16
C4	Δr	0.85±6.22	-0.84±3.26	4.32±2.92	-0.19±4.03

The table presents mean±SD of values corrected for plasma volume loss. Beep - maximal multistage 20m shuttle run test, RSA – reaped speed ability test. Δ – the difference between results: Δp = post-test – pre-test, Δr = recovery – pre-test. The analyses were performed before (baseline, pre-test) and after the effort (5 minutes post-effort and during lactate recovery time about 1 hour after the test).

It must be pointed out that this form of non-normally distributed data presentation is not suggested as it may be misleading.
